# Supplementary material for: Simultaneous Improvement of Dissolution Behavior and Oral Bioavailability of Antifungal Miconazole via Cocrystal and Salt Formation
Source: Pharmaceutics. 2022 May 22;14(5):1107. doi: 10.3390/pharmaceutics14051107 (PMC9143750; doi:10.3390/pharmaceutics14051107)
Supplement: Supplementary file 1 [file pharmaceutics-14-01107-s001.zip › pharmaceutics-1719954-supplementary.pdf]

Supporting Information
for

Simultaneous Improvement of Dissolution Behavior and Oral Bioavailability of Antifungal Miconazole via Cocrystal and Salt Formation

Ksenia V. Drozd, Alex N. Manin, Denis E. Boycov and German L. Perlovich *

G.A. Krestov Institute of Solution Chemistry of the Russian Academy of Sciences,
1 Akademicheskaya St., 153045 Ivanovo, Russia

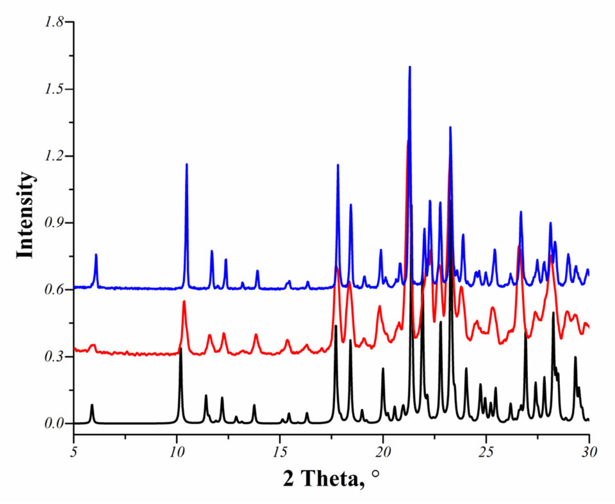

(a)

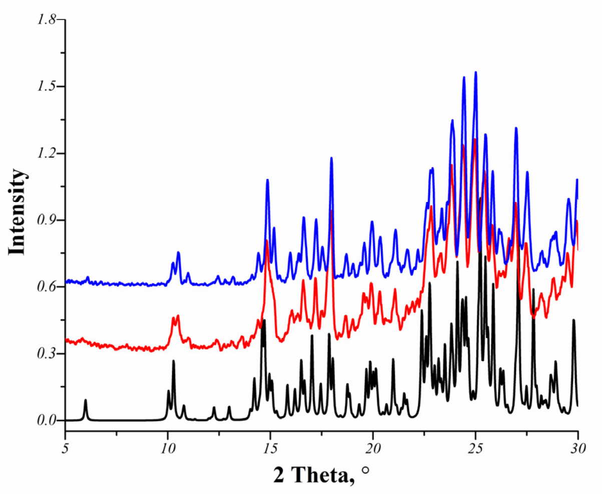

(b)

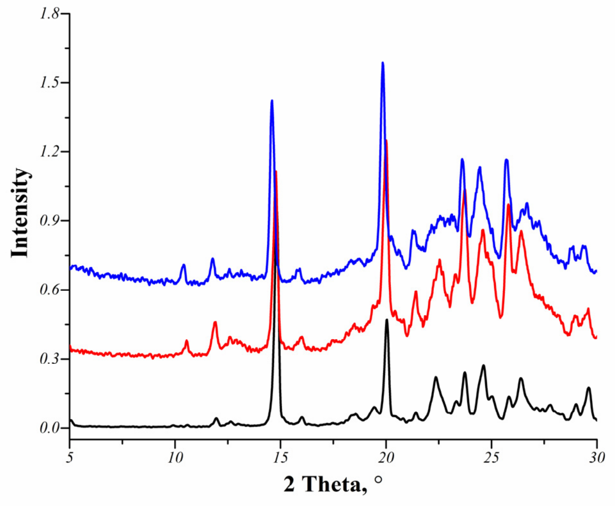

(c)

Figure S1. Overlay of the experimental PXRD patterns of the MCL multicomponent crystals prepared by LAG (red line) and slurry (blue line) and calculated PXRD (black line) form the X-ray crystal structure for: (a) [MCL+SucAc] (2:1), (b) [MCL+MlcAc] (1:1) and (c) [MCL+TartAc] (1:1).

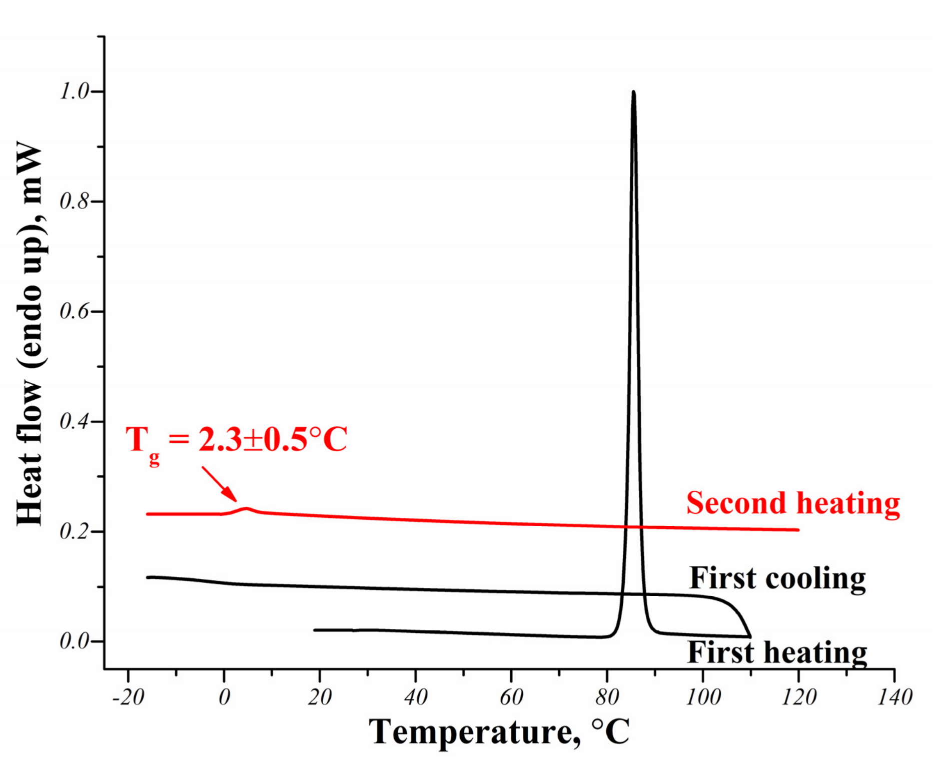

Figure S2. DSC profiles of MCL indicating its transformation from crystalline to the glassy state by cooling sample from the melt.

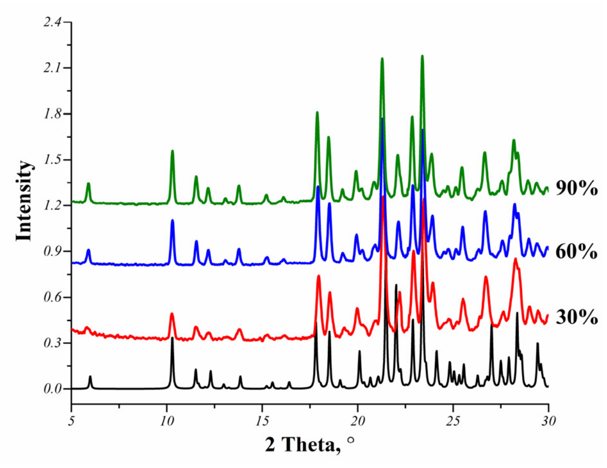

(a)

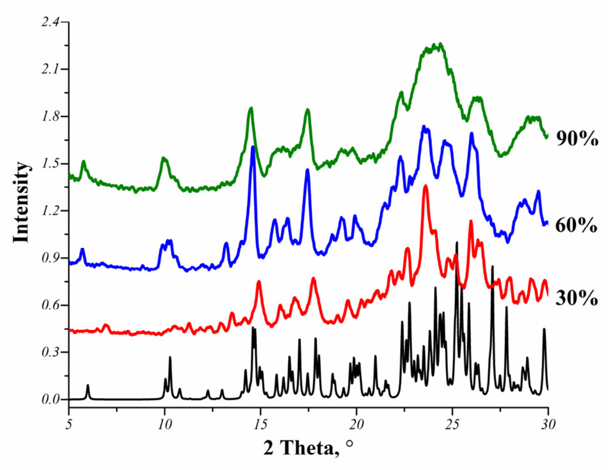

(b)

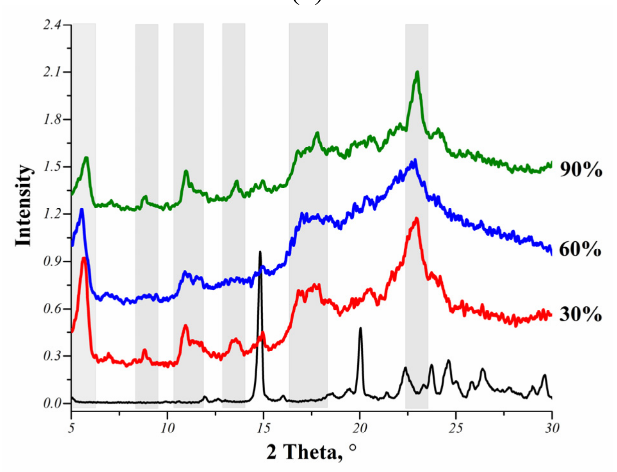

(c)

Figure S3. Overlay of the experimental PXRD patterns of the freeze-dried powders of (a) [MCL+SucAc] cocrystal (2:1), (b) [MCL+MlcAc] salt (1:1) and (c) [MCL+TartAc] salt (1:1) obtained from TBA/H_2O mixtures with different TBA content. The numbers show the percentage of TBA in the TBA/H_2O mixture. The black patterns correspond to the calculated PXRD of the MCL multicomponent crystals. Grey stripes highlight some unique peaks different from the peaks of the crystalline [MCL+TartAc] salt.

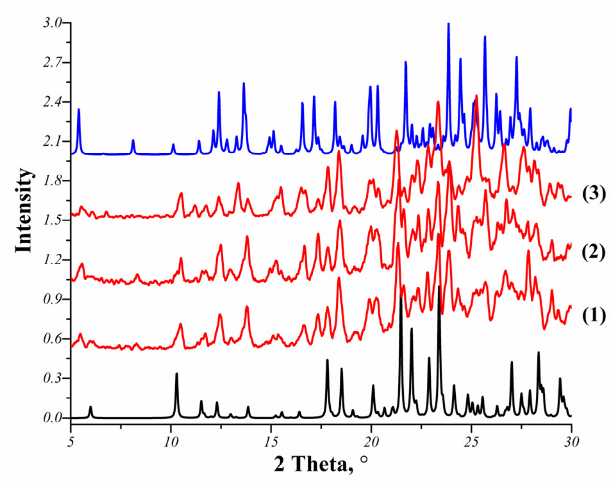

(a)

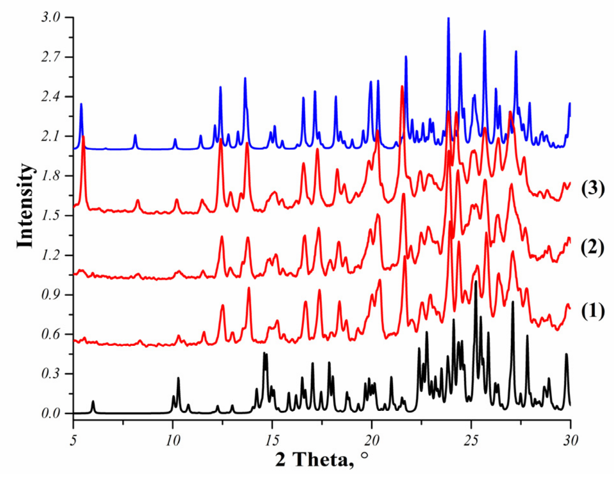

(b)

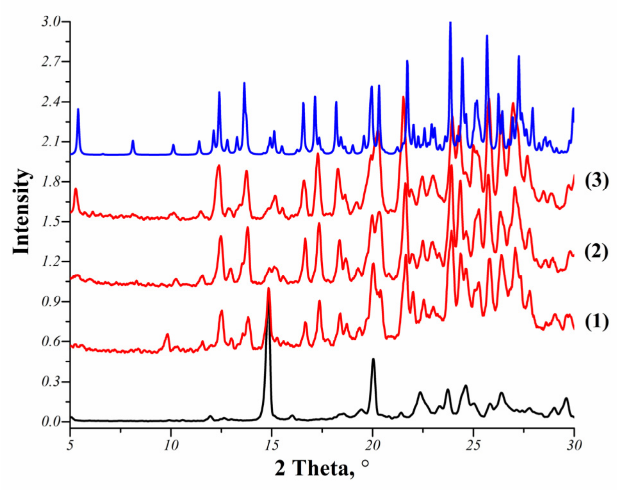

(c)

Figure S4. Results of PXRD analysis of the residual materials collected at the end of the dissolution experiments: (a) [MCL+SucAc] cocrystal (2:1), (b) [MCL+MlcAc] salt (1:1) and (c) [MCL+TartAc] salt (1:1). Key: black line – calculated PXRD of the MCL cocrystal/salt, blue line - calculated PXRD of the MCL hemihydrate. Red lines correspond to solid phases of the MCL multicomponent crystals prepared by LAG (1), slurrying (2) or freeze-drying (3) after dissolution experiments.

Table S1. Crystallinity percentage of the freeze-dried powders of the MCL multicomponent crystals calculated on basis of PXRD analysis

	Crystallinity percentage of the freeze-dried powders, %[a]		
	TBA/H_2O 30:70	TBA/H_2O 60:40	TBA/H_2O 90:10
[MCL+SucAc] (2:1)	89.9	89.7	89.1
[MCL+MlcAc] (1:1)	84.3	82.0	79.4
[MCL+TartAc] (1:1)	60.6	46.0	50.1

[a] The crystallinity was calculated from the ratio of the area of all crystalline peaks to the total area

Table S2. Thermophysical data of the MCL multicomponent crystals prepared by the multiple methods

MCL multicomponent crystal	Preparation method	T_{fus}^{onset}, °C		ΔH_{fus}^{T}, J·g[-1]	
		literature*	this work	literature*	this work
[MCL+SucAc] (2:1)	LAG	119.7±0.2	117.6±0.2	82.2±0.5	77.4±0.5
	slurry		119.5±0.2		82.5±0.5
	freeze-drying		117.7±0.2		85.6±0.5
[MCL+MlcAc] (1:1)	LAG	140.3±0.2	135.9±0.2	-	-
	slurry		140.5±0.2		-
	freeze-drying		136.8±0.2		-
[MCL+TartAc] (1:1)	LAG	171.4±0.2	170.6±0.2	164.4±0.5	163.6±0.5
	slurry		171.5±0.2		164.5±0.5
	freeze-drying		155.8±0.2		86.0±0.5

* Drozd, K. V; Manin, A.N.; Voronin, A.P.; Boycov, D.E.; Churakov, A. V; Perlovich, G.L. A combined experimental and theoretical study of miconazole salts and cocrystals: crystal structures, DFT computations, formation thermodynamics and solubility improvement. Phys. Chem. Chem. Phys. 2021, 23, 12456–12470
